# Localized Fournier's Gangrene in “End-Stage” Renal Failure: Multidisciplinary Approach and Integration of Palliative Care

**DOI:** 10.1155/2020/8868993

**Published:** 2020-11-24

**Authors:** M. Voordeckers, J. Noels, M. Brognet, M. T. Salaouatchi, M. Mesquita

**Affiliations:** ^1^Palliative Care Unit, Centre Hospitalier Universitaire, Hospital Brugmann, Brussels, Belgium; ^2^Urology Clinic, Centre Hospitalier Universitaire, Hospital Brugmann, Brussels, Belgium; ^3^Department of Internal Medecine, Clinics of Nephrology and Dialysis, Hospital Brugmann, Brussels, Belgium

## Abstract

*Presentation of the Case*. Penile gangrene is a rare entity with significant morbidity and mortality. There are only few case reports of isolated penile Fournier's gangrene in literature. Its rare occurrence, associated with complex and serious comorbidity, poses a major challenge to the attending medical personnel. A 53-year-old Caucasian patient with poorly controlled diabetes, progressive renal insufficiency, and multiple vascular complications presented with progressive necrosis of the penis (localized Fournier's gangrene). *Discussion*. Fournier's gangrene or necrotizing fasciitis refers to any synergistic necrotizing infection of the external genitalia or perineum and is a hallmark of severe systemic vascular disease. Fournier's gangrene is an absolute emergency because the time interval between diagnosis and treatment significantly influences morbidity and mortality. Despite aggressive management, the estimated mortality rates range from 57 to 71%. *Conclusions*. Improved integration of palliative care services into the care of such patients is important to improve end-of-life care even though they do not have a malignant disease. The “Palliative Care Indicator Tool” can help identifying people at risk of deteriorating health and is important to improve end-of-life care.

## 1. Introduction

Penile gangrene is a rare entity with significant morbidity and mortality. There are only few case reports of isolated penile Fournier's gangrene in literature [1]. Its rare occurrence associated with complex and serious comorbidity poses a major challenge to the attending medical personnel.

Penile gangrene can be due to an infection (infectious gangrene) or can occur because of traumatism or vascular problems such as atherosclerosis or arteriosclerosis (dry gangrene). Distinction between infectious and dry gangrene is essential since clinical management of these two subsets differs markedly [2].

Fournier's gangrene (FG) or necrotizing fasciitis refers to any synergistic necrotizing infection of the external genitalia or perineum. Predisposing factors such as diabetes, alcohol or intravenous drug abuse, cancer, obesity, advanced age, renal failure, immunodeficiency and HIV infection, and/or heart-and peripheral arteries disease can be identified in nearly all cases [3].

FG can be idiopathic, may result from perineal and genital skin infections, or can occur following genitourinary procedures or trauma. In approximately 95% of cases, a source of infection can be identified. The most common source of infection is urethral stricture, perianal abscesses or fissures, and fistulas [4].

The most frequent cause of ischemic penile gangrene is diabetes mellitus with end-stage renal disease leading to secondary hyperparathyroidism. This condition may cause calciphylaxis of penile arteries, leading to a decreased blood flow. It is important to exclude this diagnosis in uremic patients [5].

Fournier's gangrene is an absolute emergency because the time interval between diagnosis and treatment significantly influences morbidity and mortality. Ischemic gangrene of the male genitalia is a hallmark of severe systemic vascular disease. Because of poor blood flow leading to anoxia and inflammation, any ischemic lesion can become infected, leading to FG.

Foul, feculent odor with soft-tissue crepitation is the classical finding. Prompt surgical debridement is the norm and, if needed, suprapubic cystostomy should be performed. The definitive management is either partial or total penectomy depending upon the extent of involvement. A permanent perineal urethrostomy is needed in patients undergoing total penectomy. Diabetes with penile gangrene has a high mortality rate [6].

The treatment options for dry gangrene include surgical intervention or observation while awaiting autoamputation of the demarcated tissue. Patients with atherosclerosis, resulting from underlying diabetes mellitus and chronic renal failure, are predisposed to infection and often have extensive history of hospitalization. Weiner et al. noted 57% mortality rate in their seven patients within 6 months after initial presentation. Stein et al. noted 71% mortality rate in their series at 6 months of follow-up [7, 8].

In certain cases of FG, an early palliative care approach is required to improve the quality of life of the patient. We report a case of localized FG of the penis, which required different treatment options, and PC.

## 2. Case Report

### 2.1. Patient Information

A 53-year-old Caucasian (Syrian) patient, with poorly controlled diabetes mellitus type 2 and chronic renal insufficiency stage 3, was admitted to the nephrology department because of progressive kidney failure. His medical history was significant for left ischemic stroke with sequential right hemiparesis, arteritis of the lower limbs requiring amputation of the right leg, ischemic heart disease and coronary artery bypass grafting (CABG), long-standing arterial hypertension, and chronic obstructive pulmonary disease (COPD).

### 2.2. Clinical Evolution and Treatment

On the day of admission, the patient complained of pain in the penile region. A chronic indwelling urinary catheter was already in place for management of urine voiding.

A physical examination revealed a whitish liquid from the urinary meatus. The patient had a lot of pain with an estimated visual analog scale (VAS) of 8. However, because of a total language barrier, it was difficult to grasp the exact sensation. At that moment, no penile skin lesion was observed. There were no signs of inflammation or erythema. The urinary catheter seemed to provoke a painful sensation; a bladder spasm was not excluded.

Laboratory investigations at presentation were significant for serum creatinine of 4.5 mg/dL (0.7–1.2) with an estimated glomerular filtration rate of 14 ml/min/1.73 m^2^ using MDRD equation, phosphate, 1.2 mmol/L (0.75–1.39); calcium, 2.26 mg/dl (2.15–2.50); albumin, 21 g/L (40–49); parathyroid hormone, 111 ng/L (>49 for end-stage renal disease (ESRD)); vitamin D, <5 *µ*g/L (30–60); hemoglobin, 8 g/dL (13–18); HbA1c, 8.3% (4–6); and C-reactive protein (CRP), 38.5 mg/L (<5).

Urine culture grew *Staphylococcus epidermidis* (<20.000 col/ml).

The urologists suggested anticholinergics and a local antiseptic solution. Painkillers were prescribed.

**Figure 1:**
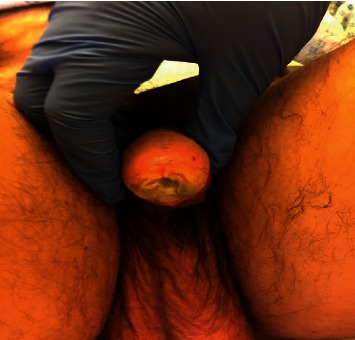
A rapidly progressing lesion appearing on the anterior part of the glans penis.

One week later, a very painful, rapidly progressing lesion appeared on the anterior part of glans penis (Figure 1). The ulcer was covered with a thick layer of fibrin. The greenish coloration was suggestive for a *Pseudomonas* infection. The ulcer could be due to a traumatic wound caused by the urinary catheter, but necrosis was not excluded. The patient became agitated and developed a fever (38°C).

The laboratory examination showed elevated CRP (341 mg/L). Hemocultures were collected, and empiric antimicrobial therapy (ceftriazone) was administered parenterally.

Despite antibiotics, the patient remained febrile, and he was malnourished (BMI 18) and septic. Antimicrobial therapy was adapted by the infectiologists in order to cover a *Pseudomonas* infection, and amoxicillin + ceftazidime was prescribed.

**Figure 2:**
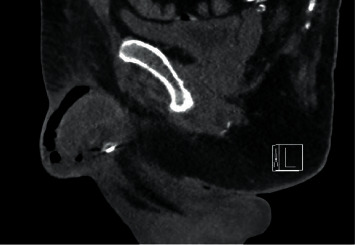
Computed tomography (CT) of the abdomen and pelvis: significant infiltration of the whole of the penis until its origin and its periphery at the pubic level, with many air bubbles within it. No infiltration of the perineum. Diffuse calcified atheromatous. Fournier gangrene located at the penis level.

Due to worsening of the patient's general condition, a computed tomography (CT) of the abdomen and pelvis was done and showed a global infiltration with air bubbles in the penile soft tissues reflecting gangrene. No air bubbles were visible inside the corpora cavernosa, and no infiltration of the perineum was seen (Figure 2).

The diagnosis of localized FG of the penis was primarily based on clinical findings. The common laboratory findings were nonspecific. CT scan helped in the diagnosis and evaluation of the extent of the disease to guide appropriate surgical treatment.

Because of extensive local necrosis, a chirurgical penile debridement was done, and 1/3 of the glans penis was amputated.

Histopathological examination revealed acute and chronic inflammation and extensive necrotic tissue with the presence of microorganisms (secondary infection), and no calcification of the arterial walls was seen. Diagnosis of isolated Fournier's penile gangrene was confirmed.

Hemocultures confirmed the infection with *Pseudomonas aeruginosa*. Antibiotics were adapted once again according to the results of blood cultures; a combination of Méropénéme-Colimycine-Metronidazole was prescribed.

**Figure 3:**
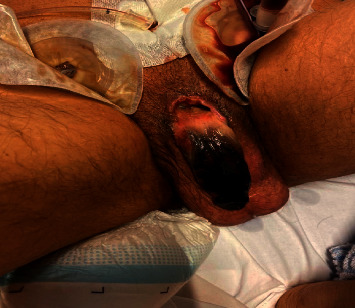
Due to progressive local necrosis, several debridations under narcosis were done, and a suprapubic catheter was placed.

Due to progressive necrosis of the penile shaft, the patient underwent subsequent debridations at the end a total resection of penile skin tissue, necrotized tissue, the ventral side of the glans, and resection of the necrotized part of the urethra. The patient and his family were informed of the risk of partial or total penectomy. A suprapubic catheter was placed during the second debridement (Figure 3).

Hyperbaric oxygen therapy was suggested, but because of his poor general condition (Karnofsky index 20) and frequent restlessness, this treatment could not be carried out.

Once more, the unfavorable clinical evolution was discussed with the patient and his family. They were told that, due to advanced renal insufficiency with diffuse arterial disease and irreversible vascular penile necrosis, amputation of the penis spontaneously or surgically was inevitable.

Daily clinical worsening of the patient's condition, mental confusion with almost complete loss of contact was noted. The patient remained often in fetal position under his blanket, and he refused intake per os. Enteral or parenteral feeding/treatment became impossible because the patient tore off gastric tubes as well as perfusion catheters.

After multidisciplinary discussion, it was decided that this patient should rapidly benefit from a palliative care approach to make this patient's end-of-life comfortable. Due to language barrier, direct communication with the patient and his family was impossible. With support of an interpreter and an intercultural mediator, a meeting was organized with the family and palliative care (PC) team to discuss the patient's pernicious prognosis and the palliative setting in order to transfer the patient to the PC unit. At first, the family was reticent to this approach despite several attempts during the past two weeks to explain the difficult and serious medical condition; they found it difficult to accept the bad outcome. After several meetings, agreement between the family and the PC team was obtained; the patient was admitted to the PC unit. He died 2 days later.

## 3. Discussion

Following the poor clinical evolution of the penile gangrene in our patient despite treatment with antibiotics and several surgical debridements, the palliative care approach was required to improve the quality of end-of-life in our patient.

### 3.1. Comfort Therapy and Palliative Care

Historically, palliative care was focused on patients with incurable cancer. However, the current view is that access to palliative care should be based on need rather than diagnosis and so, many patients with nonmalignant diseases qualify as well [9, 10].

The general aim of palliative care is improving quality of life of patients and families, to help them adjusting to progressive disease and to improve care and patient-centered outcome.

One of the most important barriers for introducing palliative care is the lack of validated and practical tools to identify patients in need.

In 2002, Belgium adopted a legal framework for taking care of life's end: “The Act of 14 June 2002 on palliative care,” which grants all citizens the right to palliative care in the context of support throughout the end-of-life.

Recently, a tool was developed to identify persons with palliative care needs in the population or in specific settings or conditions. This “Palliative Care Indicator Tool” (PICT) was included in “the Official Gazette” (het Belgisch staatsblad) on November 20, 2018 (M.B. 20/11/2018-A.R. du 21/10/2018).

This PICT is a guide to identifying people at risk of deteriorating health and dying. It has 6 general indicators of deteriorating health and clinical signs of advanced, progressive, underlying conditions. It helps professionals identify people for care planning. The first question is “would you be surprised if your patient died within the next 6 to 12 months?” This new index allows extending significantly the number of individuals admitted to palliative care. What will happen to each person and when is often uncertain? The PICT does not give a “prognosis” or a time frame [11].

Palliative care demands an active participation of patients, family, and team(s) in shared decision-making. Multidisciplinary and multidimensional approach is needed to identify values and preferences and to make an advance care planning. Good care for people with advanced illnesses is based on a person-centered, multidimensional (physical, emotional, social, and spiritual), and respectful care practiced by a competent interdisciplinary team.

### 3.2. In Our Patient Case

1. This process took several days, partly influenced by cultural differences: his family could not accept the poor clinical outcome. After repeated discussion, they understood the palliative approach and accepted a transfer to a palliative care unit.
2. His symptoms and pain were exacerbated significantly by emotional and psychological distress because of a language barrier (he only spoke Syrian). Moreover, the patient showed episodes of confusion, and direct contact with him was very difficult.

This type of complex situations emphasizes the need for multidisciplinary team involvement to address all aspects of his physical, psychological, social, and spiritual well-being [12].

Although excellent pain control was only achieved in the last days of life, a combined approach involving surgical colleagues, renal physicians, anesthetists, and palliative care physicians was essential to optimize symptom control.

Our patient's pain was exacerbated due to emotional and psychological distress because he could not communicate with us. He had been through mutilating surgery, but was not improving. It was difficult for his family to accept his condition. These situations require collaboration with interpreters and intercultural mediators.

Patients with nonmalignant diseases but life-limiting diseases, such as advanced respiratory diseases, advanced cardiac disease, advanced neurological diseases, frailty, and end-stage renal disease (ESRD), unlike patients with malignant diseases experience a low proportion of PC consultations and intense care at the end-of-life despite significant morbidity and mortality in these populations [9, 10]. This also applies to some patients with FG, whose pain is difficult to control and have poor short-time prognosis.

Family-reported quality of end-of-life care was significantly better for cancer and dementia patients than for patients with ESRD, cardiopulmonary failure, or frailty, largely due to higher rates of palliative care consultation and do-not-resuscitate orders and fewer intensive care unit (ICU) deaths among cancer and dementia patients. Increasing access to PC and goals-of-care discussion that addresses code status and preferred setting of death, particularly for patients with end-organ failure and frailty, may improve the overall quality of end-of-life care for patients dying of these illnesses [13].

Our case presentation highlights the need for improved integration of PC services into care of FG patients to improve quality of EOL care.

## 4. Conclusion

Isolated Fournier's penile gangrene is associated with significant morbidity and mortality. Despite prompt intervention, FG can rapidly evolve into progressive tissue destruction, sepsis and ultimately death as observed in our patient who had multiple comorbidities.

Pain control is very important in this situation and it demands a multidisciplinary team approach. Palliative care consultation should be programmed early to control symptoms and to support the family.

This case report highlights the need for improved integration of PC services into care for patients to improve quality of end of live (EOL) care.
